# The Influence of Attachment Styles and Personality Organization on Emotional Functioning After Childhood Trauma

**DOI:** 10.3389/fpsyt.2019.00643

**Published:** 2019-09-05

**Authors:** Jürgen Fuchshuber, Michaela Hiebler-Ragger, Adelheid Kresse, Hans-Peter Kapfhammer, Human Friedrich Unterrainer

**Affiliations:** ^1^ Center for Integrative Addiction Research (CIAR), GrünerKreis Society, Vienna, Austria; ^2^ University Clinic for Psychiatry and Psychotherapeutic Medicine, Medical University Graz, Graz, Austria; ^3^ Institute for Pathophysiology und Immunology, Medical University Graz, Graz, Austria; ^4^ Department of Religious Studies, University of Vienna, Vienna, Austria

**Keywords:** adult attachment, personality organization, structural equation modeling, childhood trauma, primary emotions, affect regulation, mediation

## Abstract

**Background:** Current literature suggests a tenuous link among childhood trauma, personality organization, adult attachment, and emotional functioning in various psychiatric disorders. However, empirical research focusing on the interaction of these concepts is sparse. Therefore, this study intends to investigate the influence of personality organization and attachment dimensions on the relationship between childhood maltreatment and emotional functioning in adult life. To assess emotional functioning, we adopted the Affective Neuroscience model of primary emotions, comprising SEEKING, FEAR, ANGER, SADNESS, CARE, and PLAY.

**Methods:** The total sample consisted of 616 nonclinical adults (Age: *M* = 30; *SD* = 9.53; 61.9% female). Path analysis was applied to investigate interactions among childhood trauma, personality organization, adult attachment, and primary emotion dispositions.

**Results:** The findings suggest that childhood trauma significantly predicted deficits in personality organization and insecure attachment (all *p* < 0.001). Furthermore, a reduced level of personality organization was significantly associated with increased ANGER (*p* < 0.001), whereas adult attachment substantially predicted primary emotion dispositions in general. Moreover, the results indicate significant mediational effects of personality organization and attachment dimensions on the relationship between childhood trauma and primary emotions (*p* < 0.01). The final model was able to explain 48% of the variance in SADNESS, 38% in PLAY, 35% in FEAR, 28% in CARE, 14% in ANGER, and 13% in SEEKING.

**Discussion:** The findings contribute to the understanding of the relationship between childhood maltreatment and impaired emotional functioning in adult life. Furthermore, the importance of personality organization and attachment dimensions for emotion regulation is underlined. Consequently, the treatment of patients with childhood trauma should focus on facilitating the development of more secure attachment patterns and increased personality functioning to improve overall emotional functioning.

## Introduction

There is considerable evidence linking childhood maltreatment to a wide range of adult psychopathology (1). In accordance with this, a recent review by Teicher and Samson (2) suggested that childhood trauma is substantially related with morphological alterations in a number of brain regions, specifically the anterior cingulate, dorsal lateral prefrontal and orbitofrontal cortexes, the corpus callosum, and the hippocampus. Furthermore, childhood trauma is linked with enhanced amygdala response to emotional cues and conflict processing as well as diminished striatal response to anticipated rewards. In this context, converging results suggest that the association between childhood trauma and adult psychopathology might be mediated by disturbances in the neurobiological development related to cognitive control and emotion regulation (3–5). Empirically, childhood trauma is often assessed by the retrospective amount of emotional, physical, and sexual abuse, as well as emotional and physical neglect and deprivation (6).

With regard to emotional functioning, Affective Neuroscience (AN) proposes a framework of interdependently connected structures composed of primary, secondary, and tertiary processes (7–9). Primary processes consist of largely subcortically located basic emotions, serving as the primary motivational system of behavior. Secondary processes are linked to the limbic system and basal ganglia. These include unconscious and conditioned behavioral traits, like personality functions, object relations, and attachment patterns. Tertiary processes are predominantly neocortically based and summarize a broad spectrum of higher order cognitive functions like mentalization, mindfulness, and spirituality. Regarding the primary process foundation of personality, AN emphasizes the importance of seven neurobiologically discrete basic emotion circuits, bridging the boundary between physiological and psychological experience (7). These include SEEKING, LUST, ANGER, FEAR, SADNESS (or PANIC/GRIEF), PLAY, and CARE. With the exception of LUST, these primary emotion systems can be measured on a language-based conscious level with the Affective Neuroscience Personality Scales (ANPS) developed by Davis, Panksepp, and Normansell (10). The clinical importance of these primary emotions is underlined by their role in a multitude of psychiatric disorders, including depression (11–13), substance use disorders (14), Internet addiction (15), and autism (16). Furthermore, a recent twin study by Melchers et al. (17) implies a significant heritability of primary emotion dispositions and emphasizes the influence of environmental factors. Recent findings by Fuchshuber et al. (12) suggested a substantial association between childhood trauma and despair, which was composed of low SEEKING and high SADNESS, as proposed by Watt and Panksepp (18) and Zellner et al. (19).

Traditionally, the development of secure attachment has been linked to the genesis of emotional functioning (20). Thereby, Bowlby (21) observed that infants who were not able to establish a secure attachment to their caregiver were at higher risk for the emergence of developmental disorders, severe depression, and delinquent behavior. In accordance with this, attachment theory assumes that the development of affect regulation is linked to the early nonverbal communication between infant and primary caregiver (22, 23). Ideally, primary caregivers perceive the nonverbal affective expressions of the infant and coregulate these through symbolic mirroring and by providing physical as well as verbal comfort. This process helps the infant to tolerate its intense and primary nonverbal emotions. The repeated experience of this process is gradually internalized by the infant, which leads to the development of a positive inner working model of the self and others. These inner working models provide an internalized secure base, which enables the individual to regulate emotions in a relatively autonomous and functional way (24). Furthermore, secure attachment helps the individual to form stable and functional relationships, allowing the individual to regulate emotions with the help of others (25). In accordance with this, a secure adult attachment style might be defined by a pattern of comfortableness with intimacy, low anxiety of being rejected and unloved, as well as the ability to depend on others and having others depending on oneself (26). However, internalized traumatic early experiences lead to corresponding inner working models and insecure attachment patterns that obstruct the functional regulation of emotions (23, 27–29).

In line with this, (9, 30) proposed internalized object relations as the building blocks of the mind. Therefore, object relations consist of self-representations and object representations and affects connecting both. Similar to the inner working model of self and others in attachment theory, in Kernberg’s view, object relations are conceptualized as influenced by early relationship experiences (9, 24). Yet, in contrast to attachment theory, Kernberg assumes that memories of early relationship experiences in adults are distorted by elements of fantasy regarding the primary caregiver. Furthermore, he emphasizes the interaction between the infants temperament and its environment (9, 31, 32). In accordance with this, the process of internalization of object relations gradually shapes mental structures and personality organization through consecutive layering sequences.

Kernberg’s (32, 33) model of personality organization differentiates among three dimensions of dysfunctioning: (1) identity diffusion, which describes deficits regarding the coherence of internalized representations of oneself and others; (2) primitive defense mechanisms, meaning the dominance of early defense formations related to splitting; and (3) reality testing, indicating the ability to separate between the internal and external world. Moreover, he suggested that personality organization might be differentiated into three broad categories termed the neurotic, borderline, and psychotic level of organization. In this context, “borderline organization,” which is conceptually related but not identical with borderline personality disorder (BPD), is linked to increased identity diffusion and predominant primitive defense mechanisms, combined with a mostly intact reality testing. In contrast, a decreased ability for reality testing is linked to a psychotic organization. However, all three concepts are theoretically interlocked and display an overall continuum of personality functioning (34). Research suggests that a low level of personality organization is associated with increased aggressive dyscontrol and negative affect as well as decreased positive affect and dysphoria (35).

It might be expected, because of similar theoretical foundations and implications, that both adult attachment and personality organization are significantly interrelated. Nonetheless, research investigating the link between self-rating measures of both concepts has been sparse and studies have been made predominantly on theoretical grounds (36–38). Most empirical studies focused on the relationship between BPD and adult attachment. Their results suggested robust associations between BPD and insecure attachment patterns (39), but they did not reveal a single attachment style specifically predicting BPD (40). Moreover, deficits within the attachment system are seen as core features of BPD (28). Regarding borderline personality organization, a study by Fischer-Kern et al. (41), which investigated links between reflective functioning (42), measured by the Adult Attachment interview (43), and personality organization, measured by the Structured Interview of Personality Organization (44), found moderate associations between deficits in reflective functioning and personality organization. In addition, Hiebler-Ragger et al. (45) reported significant correlations between borderline organization and adult attachment operationalized with self-rating measurements.

### Research Question and Hypothesis

**Figure 1:**
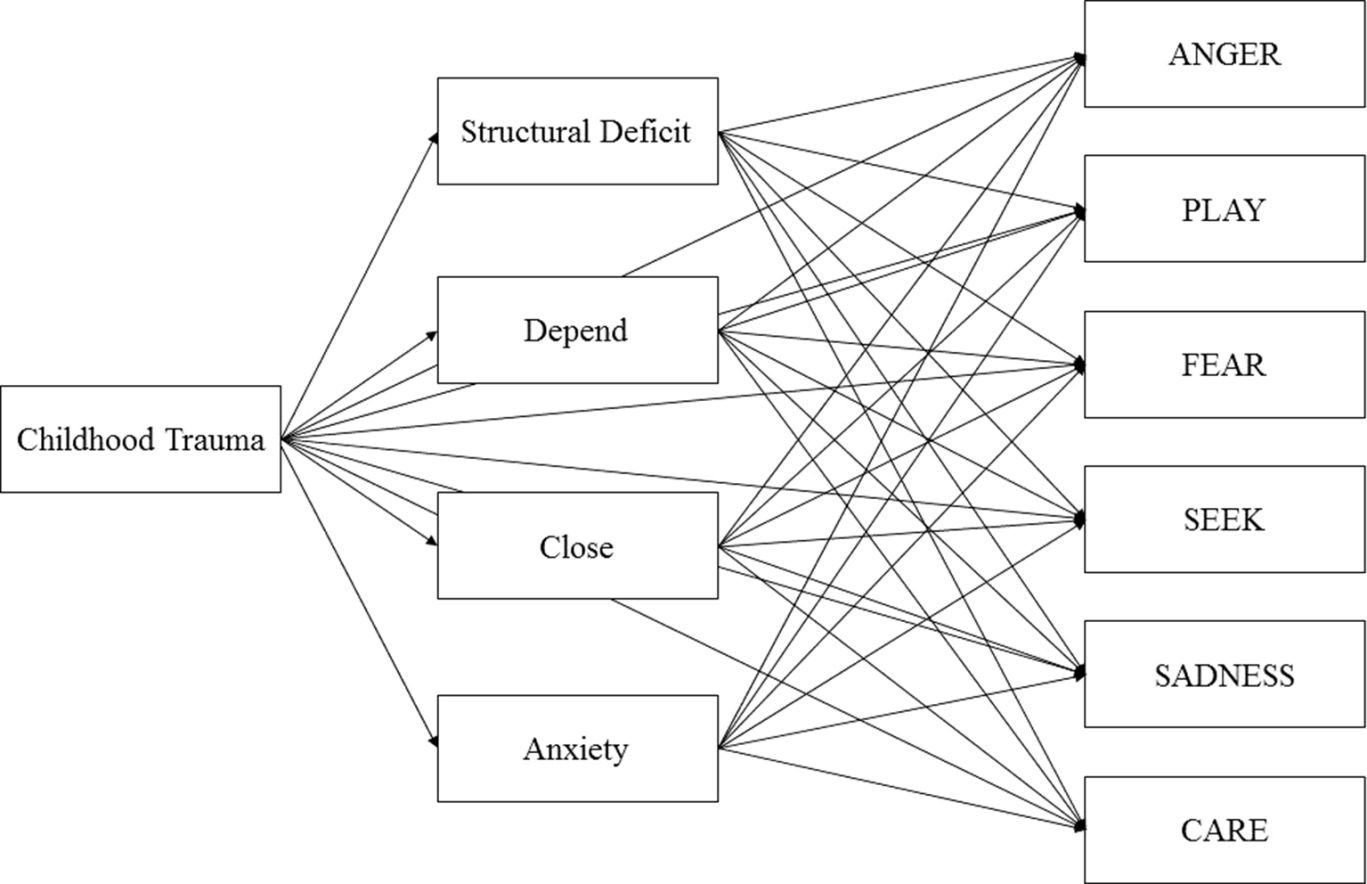
Initial model of Childhood Trauma, Structural Deficit, Adult Attachment, and Primary Emotions controlled for Age and Sex.

To map the relationship among childhood trauma, attachment, personality organization, and emotional functioning, this study applied path analysis. This statistical technique enables the investigation of simultaneous links between different concepts. Based on the research outlined above, the following hypotheses were formulated. Increased childhood trauma predicts more unsecure attachment patterns, deficits in personality organization, and decreased emotional functioning, as measured by basic emotion dispositions. Furthermore, unsecure attachment patterns and deficits in personality organization were expected to be associated with decreased emotional functioning. Therefore, we tested the hypothesis that attachment styles and personality organizations have a mediational role in the relationship between childhood trauma and emotional functioning. The conceptual framework for the hypotheses is outlined in **Figure 1**. Furthermore, we applied a multigroup path analysis approach to test if healthy participants differed from participants with a psychiatric diagnosis regarding the relationships in the path model.

## Materials and Methods

### Procedure

The sample was recruited through various social networks. Informed consent was acquired before each participant filled in the test form that included demographic questions as well as the standardized questionnaires described below. The data were acquired *via* the online-survey platform LimeSurvey^©^. Data were analyzed from all participants who were aged between 18 and 69 years, spoke German fluently, and filled in every questionnaire. Overall, 1,502 individuals responded to the online survey, however, 874 discontinued the participation before completion, whereas 12 participants did not meet the required age for participation. The study was carried out in accordance with the Declaration of Helsinki. Ethical approval was granted by the Ethics Committee of the Medical University of Graz, Graz, Austria. The recruitment of participants was carried out between 02.04.2017 and 19.03.2018.

### Psychometric Assessment

#### Childhood Trauma

The Childhood Trauma Questionnaire (CTQ) (6) [German version by Wingenfeld et al. (46)] is a 28-item self-report measure of traumatizing childhood experiences, comprising “Emotional Abuse,” “Physical Abuse,” “Sexual Abuse,” and “Emotional Neglect.” A total “Childhood Trauma” score can be calculated based on answers to the questionnaire. Because of poor reliability, the subscale “Physical Neglect” was excluded in this study (47). It employs a 1 (“never”) to 5 (“very often”) Likert scale, with higher scores indicating more severe abuse or neglect. The subscales showed good to excellent internal consistencies with Cronbach’s alpha ranging from 0.74 to 0.91. The total score exhibited an excellent internal consistency with Cronbach’s alpha of 0.93.

#### Emotional Functioning

The ANPS (10) [German version by Ref. (48); see Ref. (49), for the most recent version] is a self-report questionnaire that measures behavioral traits related to the concept of subcortical primary emotion circuits developed by Panksepp (50). Therefore, this questionnaire comprises the subscales SEEKING, SADNESS, FEAR, RAGE, CARE, and PLAY and an additional scale for “Spirituality.” It consists of, overall, 110 items, with 14 items for each subscale and is rated on a 4-point scale ranging from 1 (“strongly disagree”) to 4 (“strongly agree”). SEEKING summarizes the disposition toward feelings of positive curiosity toward new experiences, the tendency to explore, and a sense of being able to achieve relevant goals. PLAY measures the trait of being protracted toward games with physical contact, laughter, fun, as well as being generally happy and joyful. SADNESS operationalizes the tendency of feeling separation distress, loneliness, and sorrow. CARE operationalizes the individual’s tendency toward feelings of empathy, caring for children, people in need and animals, and a general enjoyment of being needed by others. FEAR measures the individuals’ tendency toward feelings of anxiety, tenseness, worries, and ruminations. ANGER is conceptualized as being easily frustrated and irritated, the frequent expression of anger in a verbal or physical way, the experience of being angry due to frustrations, and being unable to calm down. All scales showed acceptable to good internal consistencies, with Cronbach’s alpha ranging from 0.78 (SADNESS) to 0.89 (SEEKING). Because of our hypotheses, the subscale Spirituality was not analyzed in this study.

#### Personality Organization

The 16-Item Inventory of Personality Organization (IPO-16) [German version by Wingenfeld et al. (34)], is a self-report measurement of deficits within personality structure. The questionnaire is theoretically grounded in Otto Kernberg`s (32) model of personality organization. The IPO-16 is composed of three subscales: (1) “Identity Diffusion,” which measures the integrity of the representations of oneself and others; (2) Dominance of primitive defense mechanisms, such as splitting, denial, projection, and dissociation (“Primitive Defense”); and (3) the capacity to differentiate between internal and external stimuli (“Reality Testing”). A total score of structural deficits can be generated with this instrument. The items are rated on a 5-point Likert scale ranging from 1 (“never”) to 5 (“always”). Internal consistencies for the subscales were acceptable ranging from α = 0.74 to α = 0.80. The total score showed good internal consistency with a Cronbach’s alpha of 0.88.

#### Adult Attachment

The Adult Attachment Scale (AAS) (51) is a self-report questionnaire based on the assumption that early attachment experiences form relatively stable inner attachment working models that influence individual needs and behavior in later relationships (21). The AAS consists of three subscales measuring anxiety about being rejected or unloved (“Anxiety”), comfort with closeness (“Close”), and comfort with depending on others (“Depend”). The German version of the AAS (26) is composed of 15 items (five items per subscale) and is rated on a 5-point Likert scale ranging from 1 (“strongly disagree”) to 5 (“strongly agree”). Cronbach’s alpha for the subscales ranged between 0.81 and 0.87.

#### Statistical Analysis and Analysis Strategy

The path analysis and multigroup path analysis were conducted with AMOS 18. SPSS 17.0 was used for data management, descriptive statistics, and bivariate correlations, which assessed the strength of the relations among all variables. In the next step, data were fitted to an initial path model that included the following paths: Childhood Trauma to the attachment scales Depend, Anxiety, and Close; Childhood Trauma to Structural Deficit; Childhood Trauma to all primary emotions; attachment scales to all primary emotions; and Structural Deficit to all primary emotions (**Figure 1**). The model was controlled for age and sex. Furthermore, correlations between the disturbance terms of Personality Organization and attachment scales; between Depend, Anxiety, and Close; and between individual primary emotions were assigned.

After the initial model was fitted, a pruning strategy was applied in which nonsignificant paths were removed. First, nonsignificant correlations between the error terms of the individual variables were removed. Second, nonsignificant paths from Childhood Trauma to primary emotions were removed. Third, nonsignificant paths from Structural Deficit and the attachment scales to primary emotions were removed. Goodness-of-fit was assessed with a maximum likelihood estimation in AMOS. To test for mediation and indirect effects, a bootstrap was performed with a bias-corrected confidence interval of 95% and 1,000 bootstrap samples (52).

In accordance with Kline (53), the following fit indices were considered as markers for an acceptable model fit: (a) The comparative fit index (CFI) >0.90; (b) Tucker-Lewis index (TLI) relative fit index >0.90; (c) the square root error of approximation (RMSEA) <0.08 and the upper bound of its 90% confidence interval <1. For the comparison of competing models, the Bayesian information criterion (BIC) was used, with the smaller value indicating better fit.

## Results

### Sample Characteristics and Descriptive Statistics

The investigated sample consisted of 616 German-speaking adults (381 female, 61.9%). The participants ranged in age from 18 to 69 years (M = 30; SD = 9.53). A total of 231 (37.5%) participants declared a university degree as their highest educational level, 214 (34.7%) a general qualification for university entrance, 46 (7.4%) a high school degree, and 96 (15.5%) participants stated a completed apprenticeship as their highest educational level. Twenty-nine (4.7%) participants stated that they left school without graduation. Regarding the current occupation of participants, 222 (36%) were in employment, 313 (50.8%) in education, 57 (9.2%) were unemployed, and 24 (3.8%) were on pension. Concerning the current relationship status, 59 (9.6%) were married, 259 (42.0%) in a relationship, and 298 (48.4%) were single. The nationality of most participants was either German (n = 334; 54.5%), Austrian (n = 218; 35.5%), or Swiss (n = 30; 4.8%), whereas 34 (5.5%) had other nationalities. Finally, 243 (39.4%) participants declared that they had been diagnosed with a (lifetime) psychiatric disorder. The majority of these participants were diagnosed with depression (n = 147; 60%), 50 (21%) with other affective disorders, and 46 (19%) participants were diagnosed with other psychiatric disorders. As shown in **Table 1**, participants with and without a psychiatric diagnosis differed (*p* < 0.001; η^2^ = 0.03–0.15) in every examined variable, with the exception of CARE (*p* = n.s.). This included higher attachment security, less structural deficit, and less experienced childhood maltreatment in participants without psychiatric diagnosis.

**Table 1:** Descriptive statistics and differences between participants with psychiatric diagnosis (N = 243) and without (N = 373).

Measure	α	Healthy		Diagnosis		F_(1, 614)_	p	η^2^
		M	SD	M	SD			
AAS								
Depend	0.85	15.87	4.57	11.90	4.95	103.89	0.000	0.15
Close	0.87	13.18	4.82	10.30	5.17	49.83	0.000	0.08
Anxiety	0.81	11.07	4.52	13.68	5.12	44.38	0.000	0.07
IPO								
Structural Deficit	0.88	33.25	10.72	39.68	11.49	49.91	0.000	0.08
CTQ								
Childhood Trauma	0.93	32.64	12.84	42.44	16.65	67.52	0.000	0.10
ANPS								
SEEK	0.75	2.89	0.38	2.70	0.42	33.62	0.000	0.05
FEAR	0.89	2.64	0.52	3.06	0.51	96.66	0.000	0.14
ANGER	0.85	2.53	0.48	2.71	0.53	17.89	0.000	0.03
SADNESS	0.78	2.52	0.48	2.87	0.43	99.73	0.000	0.14
CARE	0.76	2.90	0.45	2.85	0.47	1.53	0.216	0.00
PLAY	0.83	2.89	0.45	2.60	0.48	57.70	0.000	0.09

AS, Adult Attachment Scales; IPO, Inventory of Personality Organization; CTQ, Childhood Trauma Questionnaire; ANPS, Affective Neuroscience Personality Scales.

As shown in **Table 2**, descriptive results suggested that the sample reported overall moderate exposure to childhood maltreatment (M = 36.50; SD = 15.22) (46). Furthermore, bivariate correlations between the examined variables suggested that the Childhood Trauma total score was significantly positively related to Structural Deficit, Anxiety, ANGER, FEAR, and SADNESS (all *p* < 0.001). Moreover, Childhood Trauma was negatively correlated with Depend, Close, SEEK, CARE, and PLAY (all *p* < 0.001) but not to sex (*p* = n.s.) (see **Table 2**). In addition, Structural Deficit was associated with every attachment scale (*p* < 0.001) and every primary emotion (*p* < 0.001) with the exception of CARE (*p* = n.s.). Finally, all attachment scales were significantly related to every primary emotion scale (*p* < 0.001).

**Table 2:** Descriptive statistics, sex differences, and correlations among examined variables.

Variable	1	2	3	4	5	6	7	8	9	10	11	12
1. Childhood Trauma	–											
2. Structural Deficit	0.37*	–										
3. Close	-0.44*	-0.48*	–									
4. Depend	-0.55*	-0.50*	0.60*	–								
5. Anxiety	0.35*	0.67*	-0.37*	-0.53*	–							
6. SEEK	-0.24*	-0.17*	0.28*	0.34*	-0.20*	–						
7. FEAR	0.25*	0.46*	-0.32*	-0.44*	0.55*	-0.33*	–					
8. ANGER	0.20*	0.34*	-0.20*	-0.32*	0.28*	-0.09	0.34*	–				
9. SADNESS	0.36*	0.51*	-0.36*	-0.54*	0.65*	-0.32*	0.73*	0.37	–			
10. CARE	-0.14*	0.08	0.28*	0.25*	0.07	0.28*	0.09	-0.06	0.06	–		
11. PLAY	-0.34*	-0.23*	0.53*	0.53*	-0.25*	0.56*	-0.39*	-0.11	-0.41*	0.41*	–	
12. Sex	0.09	-0.01	0.04	0.00	0.06	0.03	0.14*	0.06	0.15*	0.34*	0.03	–
*M* or *n*	36.50	35.79	12.04	14.30	12.10	2.81	2.81	2.60	2.66	2.88	2.78	381
*SD* or %	15.22	11.47	5.15	5.10	4.93	0.40	0.55	0.51	0.45	0.43	0.48	61.9

n = 616; *p < 0.001; Sex was coded as 0 = male; 1 = female.

### Path Analysis Regarding the Relationship Between Childhood Trauma, Structural Deficit, Adult Attachment, and Primary Emotions

An initial model proposed direct effects from Childhood Trauma to Structural Deficit, attachment dimensions, and the individual primary emotions, as well as direct effects from Structural Deficit and attachment scales to primary emotions (**Figure 1**). The model, which was corrected for age and sex, was saturated; hence, it was not possible to compute the probability level. The model was then pruned by deleting nonsignificant correlations between disturbance terms of the individual primary emotions. This included correlations between ANGER and SEEK, ANGER and CARE, and ANGER and PLAY.

This procedure yielded a model that fit the data well: RMSEA = 0.00 (90% CI: 0.00, 0.05); TLI = 1.00; CFI = 1.00; BIC = 535.63. In a further step, the model was pruned by removing nonsignificant paths. First, nonsignificant paths from Childhood Trauma to primary emotions were deleted. This included every association between Childhood Trauma and primary emotions. Second, nonsignificant paths from Structural Deficit and attachment dimensions to primary emotions were removed. This included (1) paths from Structural Deficit to SADNESS, FEAR, CARE, and SEEK; (2) paths from Close to FEAR, SADNESS, and ANGER; and (3) paths from Anxiety to ANGER, SEEK, and PLAY.

**Figure 2:**
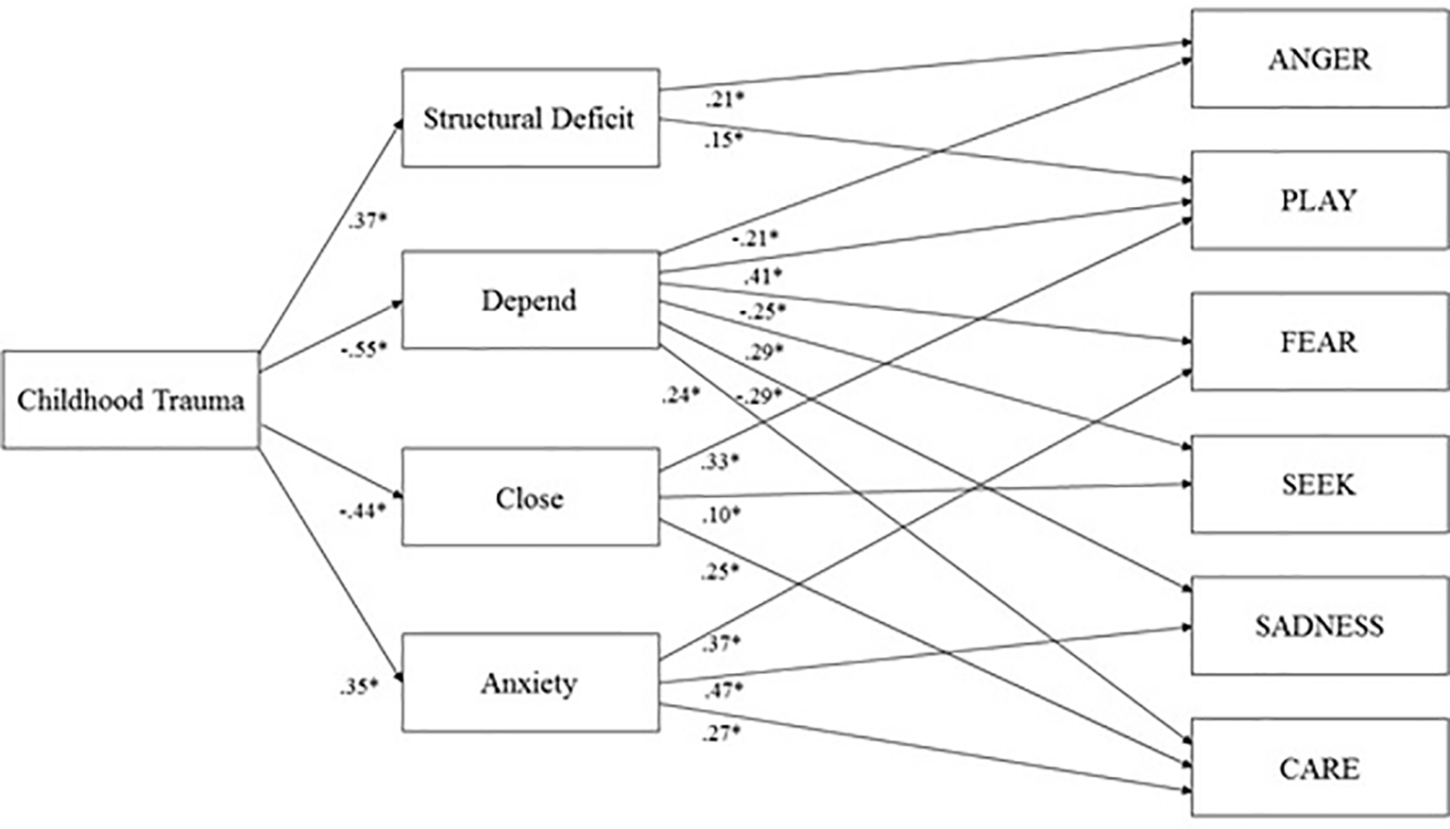
Final model of Childhood Trauma, Structural Deficit, Adult attachment, and Primary Emotions controlled for Age and Sex; *p < 0.001.

The trimmed model is presented in **Figure 2**. This model showed good fit: RMSEA = 0.03 (90% CI: 0.01, 0.05); TLI = 0.99; CFI = 1.00; BIC = 490.35. The reduction in BIC score was Δ 45, which indicated that this model was significantly more parsimonious than the initial model and therefore a better fit for the data.

### Direct Effects

As shown in **Figure 2**, this model suggested that Childhood Trauma is significantly related to Structural Deficit (β = 0.39; *p* < 0.001), Depend (β = -0.56; *p* < 0.001), Close (β = -0.44; *p* < 0.001), and Anxiety (β = 0.36; *p* < 0.001). Moreover, Structural Deficit showed a significant positive correlation with Anxiety (*r* = 0.60) and significant negative correlations with Depend (*r* = -0.38) and Close (*r* = -0.39; all *p* < 0.001). Moreover, every attachment scale was correlated with each other (*p* < 0.001). In detail, Anxiety was negatively linked to Depend (*r* = -0.43) and Close (*r* = -0.26), whereas Depend was positively linked to Close (*r* = 0.47). Furthermore, Structural Deficit was associated with ANGER (β = 0.21; *p* < 0.001) and PLAY (β = 0.12; *p* < 0.001). Meanwhile, Depend was associated with ANGER (β = -0.21; *p* < 0.001), SADNESS (β = -0.29; *p* < 0.001), SEEK (β = 0.29; *p* < 0.001), FEAR (β = -0.25; *p* < 0.001), PLAY (β = 0.41; *p* < 0.001), and CARE (β = 0.24; *p* < 0.001). In addition, Close was associated with PLAY (β = 0.33; *p* < 0.001), SEEK (β = 0.10; *p* < 0.02), and CARE (β = 0.25; *p* < 0.001). Finally, Anxiety was associated with FEAR (β = 0.37; *p* < 0.001), SADNESS (β = 0.47; *p* < 0.001) and CARE (β = 0.27; *p* < 0.001).

With regard to the control variables, female sex was positively associated with FEAR (β = 0.12; *p* < 0.001) and CARE (β = 0.33; *p* < 0.001), whereas age was negatively associated with Structural Deficit (β = -0.25; *p* < 0.001), Anxiety (β = -0.24; *p* < 0.001), FEAR (β = -0.13; *p* < 0.001), and PLAY (β = -0.13; *p* < 0.001).

In summary, this model was able to explain 14% of the variance of ANGER, 48% of SADNESS, 13% of SEEK, 35% of FEAR, 38% of PLAY, and 28% of CARE.

### Indirect Effects

Furthermore, bootstrap analysis revealed significant indirect effects of childhood trauma on primary emotions, mediated through its association with Structural Deficit and adult attachment. Significant indirect effects of Childhood Trauma include associations with CARE (β = -0.15; *p* < 0.01), mediated *via* Anxiety, Depend, and Close; SEEK (β = -0.18; *p* < 0.01), mediated by Close and Depend; ANGER (β = 0.20; *p* < 0.01), mediated by Structural Deficit and Depend; PLAY (β = -0.31; *p* < 0.01), mediated by Structural Deficit, Depend, and Close; FEAR (β = 0.29; *p* < 0.01), mediated by Structural Deficit, Depend, and Anxiety; and SADNESS (β = 0.31; *p* < 0.01), mediated by Depend and Anxiety.

### Multigroup Path Analysis

We further tested if healthy participants differed from participants with a psychiatric diagnosis regarding the relationships in the path model. The results revealed that both groups showed no significant differences in their path associations regarding the global model (恍χ²_(19)_ = 23.66; *p* = n.s.).

## Discussion

This study investigated the role of adult attachment and personality organization regarding the relationship between childhood trauma and adult life primary emotion functioning. Path analytic estimations concerning the indirect relationship between childhood trauma and primary emotions support the assumption that the influence of childhood trauma on primary emotion dispositions in adults is mediated by deficits in personality organization and insecure attachment. These results are largely in accordance with a growing field of research studies linking childhood trauma to emotion dysregulation and emotional dysfunctioning (3–5). Moreover, the results of the estimated direct effects suggest that the relationship between emotional dysfunctioning and childhood trauma might be the result of dysfunctional internalization processes related to traumatic early object relations, which lead to deficits in personality organization and insecure attachment patterns in the adult mental apparatus (9, 24). Furthermore, this study was able to gather evidence for the clinical significance of the AN-framework. In accordance with this, participants diagnosed with a psychiatric disorder not only exhibited more childhood trauma but also showed more deficits, in comparison to healthy participants, in all secondary order concepts as well as increased negative primary emotion dispositions and decreased dispositions in almost all positive primary emotions.

Our results underline the assumed importance of personality organization and adult attachment in emotional functioning proposed in psychodynamic literature (9, 20, 23, 54) and deepen the understanding of this connection. When computed within a single model, we find that structural deficit is significantly associated with increased PLAY and ANGER, whereas attachment dimensions are related to the measured primary emotion dispositions in general. More specifically, comfort with dependence on others shows several associations to decreased ANGER, FEAR, and SADNESS and increased PLAY, SEEK, and CARE. Comfort with closeness is linked with increased PLAY, SEEK, and CARE, and anxiety about being rejected or unloved predicts increased FEAR, SADNESS, and CARE. These results suggest that deficits in personality organization and insecure attachment mainly foster primary emotional traits, which are experienced as unpleasant (ANGER, FEAR, and SADNESS), whereas secure attachment predominately fosters pleasant primary emotion dispositions (SEEK, PLAY, and CARE). This is with the exception of “anxiety of being rejected,” which is linked to increased CARE, reflecting the relationship of this concept with the insecure ambivalent or preoccupied attachment style, which is characterized by excessive clinging to attachment figures (55, 56). Furthermore, the rather small relationship between deficits in personality organization and increased PLAY might be caused by a suppression effect in our path model, as correlation analysis suggested an inverse relationship between these two concepts. In summary, the relationship among attachment, personality organization, and emotional functioning might be explained, in accordance with basic assumptions of attachment and object relations theory, by the affect-integrating role of underlying internalized working models and object relations (9, 20, 54, 57).

Notably, deficits in personality organization are predominantly related to increased levels of ANGER compared to adult attachment. This result echoes Kernberg’s (9, 32, 58) conceptualization of personality organization, which (in line with Kleinian object relation theory) (59) emphasizes its crucial role in the integration and neutralization of aggressive affects. In contrast, adult attachment demonstrated stronger relations with every other facet of primary emotion dispositions, highlighting the importance of secure attachment in affect regulation and emotional functioning (20, 60).

With regard to proposed neural correlates of affect regulation, linked to the prefrontal and anterior cingulate cortex (61, 62), future studies might further investigate the functional and structural relationship between these neocortical areas and childhood trauma, adult attachment patterns, as well as personality organization. Furthermore, with regard to ANGER, which according to Panksepp (63) is mediated largely by a complex neural network, including the medial amygdala, the bed nucleus of the stria terminalis, the medial and perifornical hypothalamus, and dorsolateral parts of the periaqueductal gray, it seems plausible that the individual’s personality organization might also impact functional properties of these structures. Therefore, future studies might aim to examine the influence of therapeutic interventions directed at the improvement of personality organization based on their effect on these subcortical regions. Specifically, this might include research on neurofunctional effects of psychodynamic and attachment oriented intervention strategies like Mentalization Based Therapy (64) or Transference Focused Psychotherapy (65). Furthermore, another good example might be Mindfulness meditation techniques, which were observed to positively influence feelings of anger and anxiety in clinical patient groups (7, 66, 67). Moreover, there is a plethora of pharmacological compounds that were found to be effective in anger treatment, including mood stabilizers, serotonergic medication, and antipsychotics (68). Therefore, future research might aim to investigate the differential psychodynamic effects of psychopharmacological medications.

The direct paths of the investigated model increased the understanding of the relationship between personality organization and adult attachment. In line with previous theoretical and empirical studies (23, 36, 38, 41, 45) correlation analysis revealed substantial links between personality organization and adult attachment, which reflects conceptual similarities of both concepts. The strength of the relationships ranged from medium negative correlations with “comfort with dependence and closeness” to a large positive correlation with “anxiety of being rejected” (69). Moreover, correlation analysis revealed substantial links between emotional functioning and personality organization in addition to adult attachment. However, because of the substantial correlations between both personality organization and adult attachment, the influence of personality organization on primary emotions is diminished by adult attachment, if both concepts are considered within a single model.

The results of the multigroup analysis indicated no significant difference between healthy and diagnosed participants regarding the strength and direction of the relationship between childhood trauma, adult attachment, structural deficit, and primary emotion functioning. Therefore, this finding suggested a continuum model regarding the relationship between childhood trauma and adult personality and psychopathology.

Some limitations of this study should be noted. Despite applying path analysis, the design of this study was cross-sectional. Therefore, the investigated pathways among adult attachment, personality organization, and primary emotions cannot be seen as strictly causal. Future research might therefore conduct longitudinal studies to explore the predictive links between these concepts. Furthermore, our sample contained a rather large proportion of participants with a wide range of psychiatric disorders. This might have led to confounding effects within our model. Although multigroup analysis regarding differences between participants with and without psychiatric disorders revealed no significant difference between these groups, future work should focus on differences within the relationships between these concepts in relation to groups differing in psychopathology. Therefore, psychiatric disorders should be assessed more thoroughly by means of standardized clinical interviews. Nevertheless, it seemed reasonable for this explorative study to investigate a continuum between health and pathology. Furthermore, the attrition rate within our sample was relatively high (58%), which might suggest a certain amount of reactivity to the questions and could a have had an impact on the representativeness of the data. Moreover, the use of self-rating measures in regard to concepts, which are at least partly regarded as unconscious (61), might be seen as insufficient because they only map the conscious surface structures of these concepts. Therefore, future studies applying structured interviews should be conducted to strengthen the validity of our results. Lastly, this study did not apply measurements to assess possible self-presentation bias, hence, we cannot rule out that diminished abilities of self-reflection or tendencies toward distorted self-presentation might have influenced our results.

## Conclusion

The current study contributes to the knowledge of how childhood trauma, attachment insecurity, and deficits in personality organization influence emotional functioning. Our results suggest that both attachment and personality organization explain the association between abuse experienced in childhood and primary emotion functioning in adult life. These findings indicate that the AN-framework, assuming linked primary and higher order processes (7), might be valuable avenues to understand the pathogenic effects of childhood trauma. Therefore, this work underlines the importance of attachment and personality organization for the treatment of psychiatric disorders associated with emotional dysfunctioning (57, 70). In accordance with this, psychotherapeutic interventions might focus on traumatically damaged object relations and the restructuring of dysfunctional personality organization and attachment patterns to foster increased self-regulation and emotional functioning in patients.

## Ethics Statement

This study was carried out in accordance with the recommendations of the ethics guidelines of the Medical University of Graz. The protocol was approved by the ethics committee of the Medical University of Graz. All subjects gave written informed consent in accordance with the Declaration of Helsinki.

## Author Contributions

JF and HU conceptualized the study. JF collected, analyzed and interpreted the data. JF, HU, and MH-R drafted the manuscript. AK and H-PK critically reviewed the manuscript. All authors gave their final approval of the manuscript.

## Conflict of Interest Statement

The authors declare that the research was conducted in the absence of any commercial or financial relationships that could be construed as a potential conflict of interest.
